# Reducing meat consumption: Results from a German survey on attitudes, behaviour and willingness to change among adults

**DOI:** 10.1371/journal.pone.0328346

**Published:** 2025-08-13

**Authors:** Almut Richter, Julia Wagner, Ramona Moosburger, Gert B. M. Mensink, Julika Loss

**Affiliations:** 1 Department of Epidemiology and Health Monitoring, Robert Koch Institute, Berlin, Germany; 2 Institute for Medical Information Processing, Biometry, and Epidemiology – IBE, LMU Munich, Munich, Germany; 3 Pettenkofer School of Public Health, Munich, Germany; King's College London, UNITED KINGDOM OF GREAT BRITAIN AND NORTHERN IRELAND

## Abstract

**Background:**

Individual meat consumption in Germany has fallen slightly in recent years, but still exceeds the recommended quantities. High meat consumption has negative impacts both on human health and the environment. This study intends to identify which population groups in Germany may have already reduced their meat consumption, based on which motives, and to capture the willingness to limit future consumption.

**Methods:**

Analyses are based on representative data from 3,178 adults living in Germany, collected in a cross-sectional, standardized telephone survey (German Health Update) in 2022. Differences between population groups are identified using chi-squared tests and logistic regression models. Results are presented with 95% confidence limits.

**Results:**

72% of the population intentionally avoid meat consumption at least occasionally. About half of the population intends to eat less meat in the future or already never eat meat. 23% have no current or future intention to limit their meat consumption. Women and higher educated persons more often claim to intentionally avoid meat consumption, currently and for the future. Those who currently eat meat less often are more likely to intend further reduction compared to those who eat meat frequently. The predominant motive for reducing meat consumption is “health” followed by “climate and environmental protection” and “animal welfare”. Climate protection as a motive for reducing meat consumption is more often mentioned by young persons and women, whereas health benefits are more important for persons aged 65 years and older.

**Conclusion:**

A large proportion of the population already cuts down on meat consumption, at least occasionally. On the other hand, consumption levels in Germany are still very high. Climate and environment protection already play an important role for meat reduction, and may help leverage the transition to a more plant-based and healthier diet in the population.

## Introduction

High meat consumption is common in high- and middle-income countries. Eating red and processed meat has been linked to several diseases, including cardiovascular disease, several types of cancer and type 2 diabetes [1,2]. In addition, livestock production is associated with an increased use of antibiotics, leading to the spread of antibiotic-resistant bacteria that pose an additional threat to human health [3,4]. Meat consumption also impacts the environment and our climate. Livestock farming is responsible for an estimated 14.5% of anthropogenic greenhouse gas emissions [5]. There are clear differences between animal species. Beef is the commodity with the highest emission intensity, with an average of around 300 kg of carbon dioxide equivalents per 1 kg of protein, whereas this figure is around 50 kg or less for pork and poultry [5]. Due to the harmful effects that high meat consumption can have on both the health of individuals and the environment, reducing meat consumption, especially red meat, is an important step towards achieving more sustainable and healthier diets.

The estimated yearly consumption of meat in Germany was 52,0 kg/capita in 2022 [6]. Although consumption has decreased in recent years, coming from 60,9 kg in 2012, it is still very high [6]. According to current recommendations for a healthy diet in Germany, adults should not eat more than 300 grams of meat and sausages per week, which corresponds to around 15 kg per year [7]. The EAT-Lancet Commission has also set a similar target for maximum meat consumption in their concept of a planetary healthy diet [8].

In order to develop targeted health-promoting campaigns, and also to implement specific supporting measures in the living environment of population groups, e.g. in canteen catering, it is important to find out more about the meat consumption of different population groups, their attitudes towards meat with regard to climate change, and their willingness to reduce it and the reasons for this.

Meat consumption habits can vary greatly, depending on the country and also between population groups within the same country [9,10]. Likewise, different motives for altering dietary behaviour can have varying degrees of importance to individuals. Asking for food choices in general, a survey in Germany revealed that 96% consider the taste and 48% the price of food to be important decision criteria [11]. Furthermore, according to the literature, the predominant motives for individual reductions in meat consumption are health, climate protection, animal welfare, taste preferences, and costs [12–16]. Social norms were also found to be potentially important [12,14,15].

In recent years, studies addressed the issue of meat consumption and potential reductions, as well as the reasons for this, in various countries [17–28]. In Germany, there is little knowledge on this topic so far, and current data are particularly lacking. Studies carried out some time ago, have revealed the following: According to the population-level NEMONIT study in 2012/13, 76% of the 1,807 participants would agree to consume only two meat meals per week in the future, whereas only 17% assumed that the German population would agree to do so [29]. In two studies among convenience samples of vegans (2013 and 2014) animal welfare-related motives were reported to be the most important ones for being vegan. Most vegans were motivated by more than one reason, and health and environment-related motives were also mentioned often [30,31]. In answers to an open-ended question among adults from Rhineland-Palatinate (federal state in Germany) in 2013 no-one mentioned climate protection as an influencing factor on their willingness to reduce meat consumption, whereas health and animal welfare reasons were given frequently [32].

These studies may be outdated by now, as increased media and political attention on the worsening climate crisis has presumably increased the population’s knowledge on the causes for global warming and options for its mitigation [33]. In 2019, the EAT Lancet Commission published the “Planetary Health Diet” [8] and movements such as “Fridays for Future” [33], which started in 2018, have also raised additional awareness of the issue. Therefore, the population’s willingness to change their meat consumption, and the motives for doing so, may have changed over the last years, at least in population subgroups.

Therefore, this study aims to contribute to a better understanding of the recent meat consumption as well as attitudes towards avoiding meat consumption in Germany. Associations of age, gender, education and community size with meat consumption frequency and intentions to eat less meat are identified. Additionally, population groups that already avoid eating meat and their motives for doing so will be characterized.

## Materials and methods

### Sample

The survey was part of the “German Health Update” (GEDA, “Gesundheit in Deutschland aktuell”). GEDA is a representative cross-sectional study of the adult population living in Germany and part of the national health monitoring conducted by the Robert Koch Institute [34]. Its aim is to provide information on health status, factors influencing health and information concerning the utilisation of the health care system. Data on meat consumption, attitudes and reasons for intentionally avoiding meat consumption were collected within GEDA 22 between June and October 2022. To recruit participants, a dual-frame approach was used, contacting respondents via both landlines and mobile phones [34]. The random selection of participants from households with more than one person was done according to the so-called Swedish key [35]. In these cases, all potential participants receive the same selection probability and one person is selected at random by a computer. This person is identified by the recorded age and gender. GEDA 22 was conducted as a standardized telephone interview using a programmed questionnaire. Due to the computer-aided interviews, a high quality of the data could already be guaranteed during the data collection through automated filtering and plausibility checks. The interviews were conducted by trained staff from a market and social research institute. Subsequent data checks (e.g. data completeness) were carried out in the scientific data centre at the Robert Koch Institute according to defined standard procedures. Further information can be found in the methodology publication of the GEDA study [34]. The survey fulfils all requirements and guidelines of the Federal Data Protection Act. The survey was approved by the Federal Commissioner for Data Protection and Freedom of Information. The Charité – Universitätsmedizin Berlin assessed the ethics of the study and approved the implementation of the study (application number EA2/201/21). The participants were informed about the aims and contents of the study and about data protection. Informed consent was obtained verbally. If informed consent was given, the interviewer documented it in the database. If informed consent was not given, the interview was not conducted. Participation in the study was voluntary. The presented analysis includes 3,178 participants aged 18 years and above who live in private households in Germany.

### Variables

The original German version of the questionnaire can be found in Supplement 1.

#### Frequency of meat consumption.

The frequency of the participants’ current meat consumption was obtained with two questions: one about the consumption of beef, pork and lamb (hereafter referred to as “red meat”), and one about the consumption of poultry. These questions related only to meat consumption. The consumption of sausage was not surveyed. Both questions had six response options ranging from never/rarely to several times a day. For the analysis, these were combined into three categories (less than once a week or never; 1–2 times per week; at least 3 times per week). Furthermore, persons who have chosen the category “at least 3-4 times per week” or higher for one or both types of meat were classified as “frequent meat consumers”.

#### Avoidance of meat consumption.

**Fig 1:**
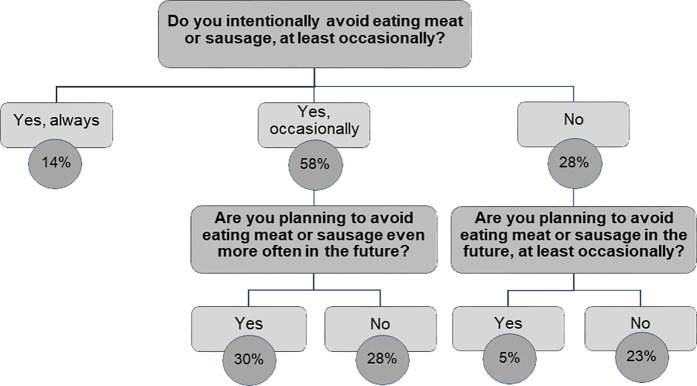
Currently intentionally avoiding eating meat and intention to limit meat consumption in the future, percentage of the population (n = 3,117).

To study the intentional avoidance of meat, a follow-up question was asked: “Do you intentionally avoid eating meat or sausage, at least occasionally?”. Answer options were “yes, always”, “yes, occasionally” and “no”. This question was followed by two conditional questions, about their intention to reduce meat consumption (more often) in the future (Fig 1). Two dichotomous variables were created for intentionally avoiding meat consumption always or at least occasionally (yes/no) and for willingness to reduce meat consumption in the future, either completely or partially (yes/no).

#### Motives for meat reduction.

Participants who already intentionally avoid eating meat, at least occasionally, were asked about their reasons for doing so. Six motives were given (multiple answers possible): “animal welfare”, “climate and environmental protection”, “health reasons”, “like to eat vegetarian food”, “don’t like the taste of meat” and “saving money”. These motives were derived from studies considering the consumption of meat [12–16] and some general motives for food choices [11].

### Covariates

For stratified analyses, several covariates were considered: gender identity (female/male), age (divided into four age groups: 18–29, 30–44, 45–64 and 65 + years) and the size of the community in which the participants live (< 5,000; 5,000 – < 20,000; 20,000 – < 100,000 and ≥ 100,000 inhabitants). Based on the information provided by the participants, three levels of education were distinguished according to the CASMIN classification (Comparative Analysis of Social Mobility in Industrial Nations) [36]: low (primary or low secondary education), medium (medium or high secondary education) and high (tertiary education).

### Statistical analysis

Frequency of meat consumption, proportion of frequent meat consumers, of people who intentionally avoid eating meat at least occasionally and of people intending to limit meat consumption (further) in the future where assessed in the total population and in subgroups. In the group of people who intentionally avoid eating meat at least occasionally, the occurrence of mentioning the given motives was determined. In order to determine the significance of the individual motives also for the whole population, all motives were set to the answer “no” for those who have not changed their meat consumption so far (and for whom the corresponding motives therefore do not apply). Differences were tested for statistical significance using chi-square tests. In logistic regression analyses (outcomes yes/no: (1) frequent meat consumption (2) intentionally avoiding meat consumption (3) intention to limit eating meat in the future) gender, age and education group were included, and odds ratios (OR) were calculated as effect estimates with 95% confidence limits (95% CI).

In order to correct for deviations in the sample from the population structure, the analyses were carried out using a weighting factor. First, design weighting was applied to account for the different selection probabilities (of mobile and landline telephone numbers). Second, an adjustment based on the official population figures was carried out with regard to age, sex, federal state and district type (as of 31 December 2019) as well as the distribution of education levels in the 2017 microcensus according to the ISCED classification [37]. Data were analysed using the survey procedures in SAS statistical software (version 9.4). A difference between groups was considered statistically significant if the corresponding p-value was < 0.05.

## Results

### Sample description

The socio-demographic characteristics of the study population are presented in Table 1. The mean age of the study population is 51.8 years (95% CI = 50.7–52.9), unweighted 58.8 years (95% CI = 58.2–59.4).

**Table 1:** Description of the study population (n = 3,178).

	n	unweighted %	weighted %*
**Gender**			
Female	1,729	54.6	51.0
Male	1,436	45.4	49.0
**Age groups (in years)**			
18 to 29	238	7.5	16.0
30 to 44	486	15.3	22.4
45 to 64	1,194	37.6	34.6
65 +	1,260	39.6	27.0
**Education**			
Low	526	16.7	27.2
Medium	1,349	42.7	52.9
High	1,283	40.6	19.9
**Community size**			
< 5,000	694	23.6	28.6
5,000 < 20,000	576	19.6	22.0
20,000 < 100,000	697	23.7	22.2
≥ 100,000	972	33.1	27.2

Missings: Gender n = 13; Education n = 20; Community size n = 239.

*The weighting accounts for the different selection probabilities of mobile and landline telephone numbers and adjusts the sample to official population figures with regard to age, sex, federal state, district type and education level.

### Frequency of meat consumption

A consumption frequency of once or twice a week is reported by 41% of the population for red meat and 50% for poultry. Meat consumption at least three times a week is reported by 26% for red meat and 12% for poultry. The remaining 32% of the population consume red meat and 38% poultry less than once a week or never (Table 2).

**Table 2:** Frequency of meat consumption (n = 3,178).

	less than once a week or never			1-2 times per week			at least 3 times per week		
	n	%	95% - CI	n	%	95% - CI	n	%	95% - CI
**red meat**	1,024	32.4	29.8 - 35.1	1,366	41.2	38.5 - 44.0	782	26.4	24.0 - 28.9
**poultry**	1,261	37.8	35.2 - 40.6	1,611	49.8	47.0 - 52.5	296	12.4	10.5 - 14.6

Missings: red meat n=6; poultry n=10.

CI = confidence interval.

According to the combined consumption frequency of red meat and poultry, 33% of the population are classified as frequent meat consumers. The remaining 67% of the population consume red meat as well as poultry less often than three to four times a week or never.

The proportion of frequent meat consumers is higher among men than among women. It is also higher in the lowest education group compared to the highest education group, and in the lowest age group compared to the highest age group. There are differences with regard to community size: Among persons in smaller communities (with less than 5,000 inhabitants), the proportion of frequent meat eaters is higher than among persons in bigger communities (with more than 100,000 inhabitants) (Table 3).

**Table 3:** Proportion of frequent meat consumers, of persons who intentionally avoid meat consumption (at least occasionally), and of persons with the intention to limit eating meat in the future, in total and by gender, age group, education group, and community size (n = 3,178).

	Frequent meat consumers			Intentionally avoid meat consumption (at least occasionally)			Intention to limit eating meat in the future		
	%	95% - CI	p-value	%	95% - CI	p-value	%	95% - CI	p-value
**Total**	**33.1**	30.5 - 35.8		**72.1**	69.3 - 74.7		**49.4**	46.6 - 52.2	
**Gender**									
Female	**22.5**	19.5 - 25.8		**77.5**	73.9 - 80.7		**55.9**	52.2 - 59.6	
Male	**44.4**	40.2 - 48.5	<.0001*	**66.2**	61.9 - 70.2	<.0001*	**42.2**	38.2 - 46.4	<.0001*
**Age group**									
18 to 29 years	**39.7**	31.6 - 48.3		**65.6**	56.7 - 73.5		**58.8**	50.1 - 66.9	
30 to 44 years	**37.9**	31.9 - 44.4		**71.8**	65.4 - 77.5		**49.8**	43.3 - 56.3	
45 to 64 years	**31.4**	27.3 - 35.8		**72.1**	67.5 - 76.4		**46.5**	42.1 - 50.9	
65 + years	**27.3**	23.5 - 31.6	0.0112*	**76.0**	72.0 - 79.6	0.1322	**47.4**	43.1 - 51.8	0.0445*
**Education**									
Low	**37.8**	32.0 - 44.0		**65.0**	58.7 - 70.8		**40.8**	35.0 - 46.8	
medium	**32.8**	29.2 - 36.7		**71.5**	67.5 - 75.1		**51.5**	47.4 - 55.5	
high	**27.8**	24.5 - 31.4	0.0263*	**83.0**	80.0 - 85.7	<.0001*	**55.0**	51.0 - 58.9	0.0004*
**Community size**									
< 5,000	**37.4**	31.9 - 43.1		**75.2**	69.6 - 80.1		**50.1**	44.3 - 56.0	
5,000 < 20,000	**34.4**	28.4 - 40.9		**72.1**	65.6 - 77.8		**50.0**	43.9 - 56.2	
20,000 < 100,000	**28.5**	23.8 - 33.9		**72.6**	66.6 - 78.0		**51.1**	45.3 - 56.9	
≥ 100,000	**28.0**	23.4 - 33.0	0.0351*	**74.5**	69.4 - 79.0	0.8438	**49.8**	44.5 - 55.1	0.9913

* *p < 0.05 in chi-square test.*

CI = confidence interval.

The logistic regression analysis shows a 2.8 higher odds ratio for men than for women to be a frequent meat consumer. In addition, persons aged 29 years and younger are more likely to be frequent meat consumers than those aged 65 years and older. Persons in the lowest education group are more often frequent meat consumers than those in the highest education group and persons living in communities with up to 5,000 inhabitants are more likely to be frequent meat consumers than those living in communities with more than 20,000 inhabitants (Table 4).

**Table 4:** Logistic regression analysis for being a frequent meat consumer, for intentionally avoiding meat consumption (at least occasionally) and for having the intention to limit eating meat in the future, odds ratios by gender, age group, education, and community size (n = 3,178).

	Frequent meat consumer			Intentionally avoid meat consumption (at least occasionally)			Intention to limit eating meat in the future		
	OR	95% - CI	p-value	OR	95% - CI	p-value	OR	95% - CI	p-value
**Gender**									
Female^1^	**1.0**			**1.0**			**1.0**		
Male	**2.8**	2.2 - 3.7	<.0001*	**0.6**	0.4 - 0.8	0.0007*	**0.6**	0.5 - 0.7	<.0001*
**Age group**									
18 to 29 years^1^	**1.0**			**1.0**			**1.0**		
30 to 44 years	**0.9**	0.6 - 1.5	0.7799	**1.2**	0.7 - 2.1	0.4565	**0.8**	0.5 - 1.2	0.263
45 to 64 years	**0.7**	0.4 - 1.0	0.0779	**1.4**	0.8 - 2.2	0.2029	**0.7**	0.4 - 1.0	0.069
65 + years	**0.5**	0.3 - 0.9	0.0146*	**1.6**	1.0 - 2.6	0.0678	**0.7**	0.4 - 1.1	0.1115
**Education**									
Low^1^	**1.0**			**1.0**			**1.0**		
medium	**0.7**	0.5 - 1.1	0.0955	**1.6**	1.1 - 2.3	0.0232*	**1.5**	1.1 - 2.1	0.014*
high	**0.6**	0.4 - 0.9	0.0057*	**2.8**	1.9 - 4.1	<.0001*	**1.8**	1.3 - 2.6	0.0005*
**Community size**									
< 5,000^1^	**1.0**			**1.0**			**1.0**		
5,000 < 20,000	**0.9**	0.6 - 1.3	0.5016	**0.8**	0.6 - 1.3	0.3933	**1.0**	0.7 - 1.4	0.9657
20,000 < 100,000	**0.6**	0.4 - 0.9	0.0088*	**0.9**	0.6 - 1.3	0.4736	**1.1**	0.8 - 1.5	0.6918
≥ 100,000	**0.6**	0.4 - 0.8	0.0024*	**0.9**	0.6 - 1.4	0.6998	**1.0**	0.7 - 1.3	0.7603

^1^reference category.

* p < 0.05.

OR = odds ratio, CI = confidence interval.

### Avoidance of meat consumption

Overall, 72% of the population report that they intentionally avoid meat intake at least occasionally, including 14% who always abstain from eating meat. Significant differences were found between gender and education groups (Table 3). Results of the logistic regression show that women are more likely than men to intentionally avoid eating meat. Persons in the highest education group are 2.8 times more likely to intentionally avoid meat consumption than those in the lowest education group. The proportion of those who intentionally avoid meat consumption does not differ by age groups or community size (Table 4).

The proportion of those who always abstain from eating meat is 14% in total (Fig 1), it is higher for women (16%) than for men (10%) and for higher education groups (8%, 14%, 17%).

### Intention to limit meat consumption in the future

Depending on whether persons already avoid eating meat always, occasionally or never, respondents were asked whether they intended to reduce their meat consumption (even more) in the future (Fig 1). A total of 30% state that they already occasionally avoid meat consumption and intend to do so even more in the future. Another 5% have not yet reduced their meat consumption but want to do so in the future. 14% always abstain from eating meat. In total, 49% of the population state that they want to reduce their meat intake in the future, either completely or partially, assuming that those who always abstain from meat already, will continue to do so in the future. It is more likely for women than for men and for the middle or highest education group than for the lowest to belong to this group (Table 4). Overall, 51% of the population do not intend to limit their meat consumption (more often) in the future.

**Fig 2:**
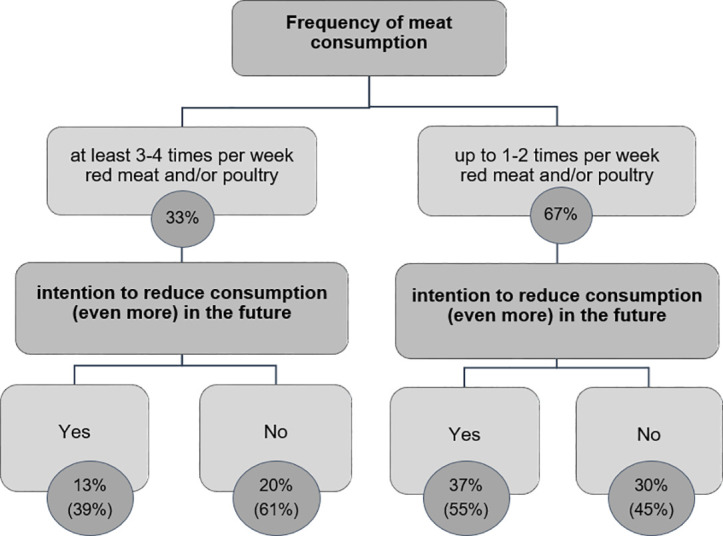
Intention to limit meat consumption in the future depending on the current frequency of meat consumption, Percentage of the population (n = 3,178). In brackets: percentage within the subgroups in terms of the frequency of meat consumption.

The intention to limit meat consumption in the future was also analyzed in the context of current frequency of meat consumption (Fig 2). Among persons who eat meat less often or never (up to 1–2 times per week red meat and/or poultry), 55% intend to eat less (or no) meat in the future, which is 37% of the total population. Among frequent meat consumers, 39% (which is 13% of the total population) state that they want to eat less meat in the future (Fig 2).

### Motives for meat reduction

**Fig 3:**
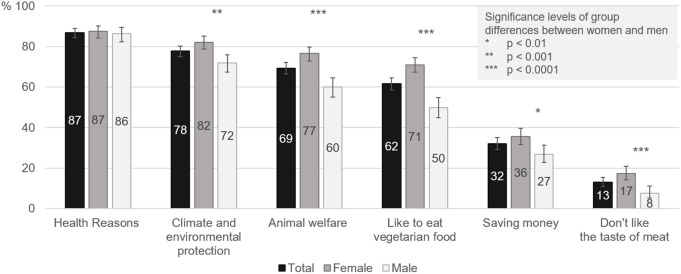
Motives for the reduction of meat consumption, shown in total (n = 2,438) and stratified by gender (female = 1,409; male = 1,018).

Those who already intentionally avoid meat consumption, at least occasionally, were offered different options for their reasons for doing so. On average, three of the six given motives were selected. “Health reasons” is the most often chosen motive for avoiding meat consumption (87%, Fig 3), followed by “climate and environmental protection” (78%) and “animal welfare” (69%). With the exception of “health reasons”, there are significant gender differences for all motives, with women choosing these motives more frequently.

The motive “health reasons” is chosen more often by older persons than by younger persons. The most frequent motive for the youngest age group is “climate and environmental protection”. Younger persons more often choose “animal welfare” and “like to eat vegetarian food” than older persons. Persons in the highest education group are also more likely to choose “animal welfare” and “like to eat vegetarian food” than those in the lowest education group, for whom “saving money” is more important (Table 5).

**Table 5:** Motives for the reduction of meat consumption stratified by age and education groups (n = 2,438).

	Health reasons			Climate and environmental protection			Animal welfare		
	%	95% - CI	p-value	%	95% - CI	p-value	%	95% - CI	p-value
**Age group (in years)**									
18–29	74.2	64.4 - 82.0	<.0001*	88.0	80.0 - 93.0	0.0064*	81.1	71.5 - 88.0	0.0075*
30–44	84.5	78.3 - 89.2		72.9	65.6 - 79.1		71.6	64.3 - 77.9	
45–64	89.5	86.1 - 92.1		75.0	70.6 - 79.0		65.8	61.0 - 70.3	
65 +	92.3	89.0 - 94.7		79.4	75.2 - 83.0		65.3	60.6 - 69.7	
**Education**									
Low	90.9	86.5 - 94.0	0.0837	80.4	74.6 - 85.1	0.2022	62.1	55.1 - 68.5	0.0131*
Medium	85.7	81.8 - 88.9		75.6	71.4 - 79.4		71.5	67.2 - 75.4	
High	85.1	81.0 - 88.4		79.6	75.8 - 82.9		71.5	67.7 - 75.2	
	**Like to eat vegetarian food**			**Saving money**			**Don’t like the taste of meat**		
	**%**	**95% - CI**	**p-value**	**%**	**95% - CI**	**p-value**	**%**	**95% - CI**	**p-value**
**Age group (in years)**									
18 to 29	72.4	63.1 - 80.1	<.0001*	35.7	27.1 - 45.4	0.0621	16.6	9.9 - 26.6	0.0606
30 to 44	69.4	62.0 - 75.8		34.9	28.0 - 42.4		8.8	5.6 - 13.6	
45 to 64	60.8	56.0 - 65.5		26.2	22.2 - 30.8		11.3	8.2 - 15.5	
65 +	50.6	45.6 - 55.5		35.1	30.3 - 40.2		16.6	13.1 - 20.7	
**Education**									
Low	51.3	44.3 - 58.2	<.0001*	38.2	31.7 - 45.1	0.0018*	14.1	10.0 - 19.6	0.1351
Medium	62.7	58.2 - 67.0		32.7	28.5 - 37.2		14.2	11.0 - 18.0	
High	70.3	66.3 - 74.0		23.6	19.9 - 27.8		9.3	7.1 - 12.1	

* significant difference in chi-square test.

The ranking of the motives differs between those who always abstain from eating meat and those who only do so occasionally. Those who always abstain from meat choose “don’t like the taste of meat” (32% compared to 9%), “I like to eat vegetarian food” (80% compared to 57%) and “animal welfare” (81% compared to 66%) more often than those who only occasionally avoid meat consumption.

Referring to the whole population, including those who have not yet changed their meat consumption, the following proportions of individual motives were observed: 63% of the population have changed their meat consumption for “health reasons”, 56% because of “climate and environmental protection”, 50% because of “animal welfare”, 44% because they “like to eat vegetarian food”, 23% to “save money” and 9% because they “don’t like the taste of meat” (multiple answers were possible).

## Discussion

A large proportion of the German population states that they intentionally cut out meat from their diet at least occasionally (72%). In addition, one in two would like to (further) limit their meat consumption in the future or already completely abstains from eating meat. However, one in five persons is currently a frequent consumer of red meat and/or poultry and does not intend to eat less meat in the future. The proportion of frequent meat consumers is higher among men, younger persons, those in lower education groups and those who live in smaller communities. Current and intended reduction of meat consumption is reported more often by women and those with higher education. Among the different reasons for eating less meat, “health reasons“, “climate and environmental protection“ and “animal welfare“ were the most chosen motives, with “climate and environmental protection” being the motive given most often by those of a younger age group.

This study provides useful information for possible measures to reduce meat consumption in Germany, which contributes to the protection of both individual health and the climate. More than 60% of the population chose “health“ and more than 50% chose “climate and environmental protection“ as reasons for cutting out meat. This implies that a substantial part of the population is already aware of the negative effects of high meat consumption on health and the climate. Other studies have shown that those who are aware of the climate impact of meat production [22,24] and believe that reducing meat consumption is an effective way to combat climate change [20,38,39], are more willing to reduce their own meat consumption. However, around 30% of the population do not yet avoid eating meat, even occasionally, and would therefore be a potential target group for information on the benefits of eating less meat, which should be aimed particularly at men and persons with a lower education background.

Studies also confirm that those who currently eat less meat are more likely to reduce their meat consumption than those who eat high amounts of meat [16,20,21,26,38,40]. On the one hand, it might be beneficial to address infrequent meat consumers, because they have, on average, a greater willingness to (further) reduce their meat consumption, so it could be easier to achieve changes in this group. On the other hand, however, the effect on public health would be greater if people with high meat consumption start reducing meat, even if they are harder to reach or to convince.

It is not necessary to reject meat completely in order to have a positive impact on both personal health and climate change. Even a reduction could bring significant benefits. In our survey, a large proportion of respondents reported that they consider a reduction in their meat intake. This high level of willingness to reduce meat consumption is in contrast with the current high levels of consumption, averaging about one kilogram per person per week [6], while a maximum of 300 g per week is recommended [7]. The amount of consumed meat has declined somewhat in recent years, while data from agricultural statistics indicate that poultry is now consumed slightly more and red meat slightly less [6], which is a positive trend in terms of health, but also considering greenhouse gas emissions.

Nonetheless, in our study, the group of 18- to 29-year-olds showed a particularly high proportion of frequent meat eaters. This corresponds to the findings of the German government’s 2024 Nutrition Report. Here too, 14 to 29-year-olds were the age group that most frequently consumed meat on a daily basis (26%) [41]. However, almost 60% of 18–29-year olds would like to limit their meat consumption in the future. So, this group presents a great potential for change. But, in order to gain a better insight into the consumption habits of the different age groups, it would be important to also determine the quantities consumed.

Our multivariate analysis revealed that although there is an association between current consumption frequency and age, there is no difference between the age groups in terms of current reduction and intention to limit meat consumption in the future.

At present, men eat meat more frequently than women, are less likely to already reduce their meat intake and are less likely to intend to reduce their meat intake in the future. Eating meat is often associated with masculinity [42,43]. Adoption of traditional gender roles may play a role in men’s meat consumption [44]. Similar to the GEDA 22 study, several other studies have found that women are more likely to be willing to reduce their meat consumption [16,20,22,24,39,45,46].

Consistent with our findings on motives for reducing meat consumption, other studies have often identified health benefits, animal welfare, and climate protection as key motives [12–15,47–49]. Other aspects such as taste and costs were also mentioned [12–14,48]. However, motives may differ between meat reducers and meat abstainers. For example, some studies suggest that animal welfare is more important to vegetarians and vegans than to meat reducers [13,14]. In a previous German study, animal welfare was also named by the majority of the population as an important criterion for making food decisions during grocery shopping [41]. However, the predominant form of livestock breeding in Germany is industrial factory farming. For example, only 1% of pig holdings have access to an outdoor run [50].

In recent years, meat consumption was also studied in other European countries, particularly in connection with climate protection aspects and the willingness to change consumption behaviour [17–28]. An online study in 2021 among adult German meat eaters (consuming meat at least once a week) found also a high level of willingness to reduce meat consumption. When asked for the level of agreement to the statement “In general, I can imagine reducing my meat consumption.” (with a response scale from 1 = strongly disagree to 7 = strongly agree), the mean response was 4.68 (SD = 1.83) [25]. This example also illustrates the challenges inherent in comparing the results of different studies. This is particularly the case if the sample was restricted to a specific group (e.g. meat consumers) or if different question formulations or response scales were used. In addition, the survey modes may differ, which can lead to differences in response behaviour [51]. Overall, the previous studies indicate that the link between meat consumption and climate change is known, at least in parts of the population and some people have already adapted their consumption habits [17–28]. Therefore, it seems useful to continue monitoring these changes.

### Implications for policy and practice

The high prevalence of respondents who already reduce meat in their diet, or plan to do so, probably reflects that people have become more critically aware that meat consumption has its downsides, considering animal welfare issues, the increasing evidence that long-term consumption of high amounts of red meat may increase various health risks [52] and/or the detrimental effect on global warming. This is also reflected in the fact that “reducing meat” is now labelled with a specific term, “flexitarian diet”. A flexitarian usually describes a semi-vegetarian or a “meat reducer” [53], although there is no universal definition for the term flexitarian [53]. Flexitarians can include individuals who consume smaller quantities of meat compared to a standard Western Diet, as well as those who only eat meat on occasion [54].

It is well known that intentions to reduce the consumption of unhealthy food do not necessarily translate into actual actions (intention-to-action gap) [55,56]. The process of altering dietary habits is complex, and barriers in everyday life need to be overcome. Cravings for meat, a lack of availability of acceptable non-meat options, non-routine meal occasions e.g. when eating out, or social influence can be challenging for many persons who intend to eat less meat [57].

Increasing the availability of attractive plant-based menus in canteens and restaurants can support individuals in their efforts to reduce meat intake. Courses on preparing vegetarian food [47] and promoting vegetarian recipes could provide further support [10]. Helping people prepare tastier non-meat dishes can make vegetarian dishes more popular. Concerns about the nutrient content of plant-based diets should be addressed by sharing knowledge about alternative protein sources and their health and climate benefits. The Food Based Dietary Guidelines in Germany were recently revised, and now also take greater account of climate protection by emphasising the importance of a plant-based diet [7]. It is necessary that these recommendations are communicated to consumers.

Emotions, cognitive dissonances (between knowledge, values and actual behaviour) and socio-cultural factors (e.g. social norms) also play a role in dietary behaviour [12,58]. However, research concerning social norms interventions for reducing meat intake is scarce [47]. Future studies should explore current barriers to a more plant-based diet, which could provide further starting points for changes.

From a health perspective, frequent meat eaters would particularly benefit from limiting meat intake. Our results show that this group has a lower intention to reduce meat consumption and they are probably more difficult to reach or may even resent appeals to reduce meat consumption, as they feel restricted in their freedom. Such behaviour is referred to as psychological reactance [59]. Therefore, an approach that is tailored to the particular stage of change of a person may be most effective. Similarly, global warming is viewed from a variety of perspectives, with some dismissing the danger, and others being highly concerned and motivated to take action. Accounting for these diverse perspectives by using different messages is important for successful audience engagement [60].

Questions about meat substitutes were not part of this study. The demand for plant-based foods and meat alternatives has grown strongly in recent years and is expected to continue [61]. However, many meat-free products that the food industry launches have been highly processed, and the impact of these ultra-processed foods on health and climate is complex and not fully understood at present. Future research could investigate whether persons who reduce their meat consumption replace meat with meat alternatives and, if so, in what proportions and with what types of products.

### Strengths & limitations

A major strength of this study is that the sample is representative for the adult population in Germany. Participants were recruited using a randomized sampling procedure. Nevertheless, a selection bias cannot be completely ruled out. The willingness to participate in health-related telephone surveys may be higher among health-conscious persons. Furthermore, persons aged 65 years and older were overrepresented, while the youngest age group was underrepresented. This circumstance is counteracted by using a weighting factor. In addition to a possible selection bias, it is also possible that response behaviour is influenced by social desirability, which could, for example, lead to a slightly too optimistic estimation of the proportion of the population who actually want to change their consumption behaviour [51]. In addition, the present analysis is based on cross-sectional data, which limits causal interpretations. The range of reasons for avoiding meat included in the questionnaire was based on important motives that have been identified in previous studies [13–17]. It is possible that further – e.g. novel, or culturally specific – relevant motives were missing from this predefined list. Other studies for example have found that “weight control” is also a motive for reducing meat consumption [13,14]. In addition to the consumption quantities estimated from agricultural statistics, there is currently hardly any data on meat consumption in Germany, especially regarding the amounts consumed by individual population groups. The consumption frequencies recorded here for red meat and poultry do not allow conclusions to be drawn about consumption quantities, as portion sizes and the consumption of sausages were not recorded. For our analysis, it was sufficient for a rough classification of intake, as another German study found that persons who report to eat meat, almost always (97%) eat sausages also [29]. The survey did not enable us to quantify the rate or amount of meat reduction either. Quantifying the scale of one’s individual meat reduction would indeed be challenging, as the term “reducing meat” is not clearly defined. We focused instead on “reducing meat” as a personal stance and attitude, which could be linked to motives, meat consumption, and future dietary intentions.

## Conclusion

In Germany, 72% of the adult population intentionally avoid meat in their diets, at least occasionally. Every second person intends to (further) reduce their current meat consumption or already abstains from eating meat completely. This indicates a broad awareness of the benefits of reducing meat intake. The most prevailing motive for reducing meat consumption is “health“, followed by “climate and environmental protection“and “animal welfare“. While willingness is a first step towards behaviour change, actually altering one’s eating habits and dietary patterns in everyday life is challenging. Barriers for change should be identified and addressed in future interventions, as meat consumption levels in Germany are still very high. In particular, supporting younger adults with a high willingness to reduce their meat consumption seems promising. A possible starting point is the food environment, for example in canteens and restaurants. Our findings provide a basis for more specific research and the development of tailored measures to promote meat reduction and emphasise climate protection as a motivator.

## Supporting information

S1 Reducing meat consumption: results from a German survey on attitudes, behaviour and willingness to change among adults. (PDF)
