# Supplementary material for: Reducing meat consumption: Results from a German survey on attitudes, behaviour and willingness to change among adults
Source: PLoS One. 2025 Aug 13;20(8):e0328346. doi: 10.1371/journal.pone.0328346 (PMC12349092; doi:10.1371/journal.pone.0328346)
Supplement: S1 — (PDF) [file pone.0328346.s001.pdf]

Supplement 1

Reducing meat consumption: results from a German survey on attitudes, behaviour and willingness to change among adults

Almut Richter, Julia Wagner, Ramona Moosburger, Gert B.M. Mensink, Julika Loss

1. **Wie oft essen Sie Rind-, Schweine- oder Lammfleisch? Nicht gemeint sind Geflügelfleisch und Wurstwaren.**

   ☐ Nie oder sehr selten
   ☐ 1-2 mal pro Woche
   ☐ 3-4 mal pro Woche
   ☐ 5-6 mal pro Woche
   ☐ Täglich
   ☐ Mehrmals täglich

2. **Wie oft essen Sie Geflügelfleisch? Nicht gemeint ist Geflügelwurst.**

   ☐ Nie oder sehr selten
   ☐ 1-2 mal pro Woche
   ☐ 3-4 mal pro Woche
   ☐ 5-6 mal pro Woche
   ☐ Täglich
   ☐ Mehrmals täglich

3. **Verzichten Sie bewusst, zumindest gelegentlich, auf Fleisch- oder Wurstkonsum?**

   ☐ Ja, gelegentlich → weiter mit Frage 4
   ☐ Ja, immer → weiter mit Frage 6
   ☐ Nein → weiter mit Frage 5.

4. **Haben Sie in Zukunft vor noch häufiger auf Fleisch oder Wurst zu verzichten?**

   ☐ Ja ☐ Nein → 6.

5. **Haben Sie in Zukunft vor, zumindest gelegentlich, auf Fleisch oder Wurst zu verzichten?**

   ☐ Ja ☐ Nein

6. Was sind Ihre Gründe für einen Verzicht auf Fleisch- oder Wurstkonsum?

Ich möchte weniger Tierleid verursachen

☐ Ja ☐ Nein

Ich möchte Klima und Umwelt schützen

☐ Ja ☐ Nein

Ich möchte etwas für meine Gesundheit tun

☐ Ja ☐ Nein

Ich esse gern vegetarische Gerichte

☐ Ja ☐ Nein

Mir schmeckt Fleisch nicht

☐ Ja ☐ Nein

Ich spare dadurch Geld

☐ Ja ☐ Nein
